# Mfge8 attenuates human gastric antrum smooth muscle contractions

**DOI:** 10.1007/s10974-021-09604-y

**Published:** 2021-06-03

**Authors:** Wen Li, Ashley Olseen, Yeming Xie, Cristina Alexandru, Andrew Outland, Angela F. Herrera, Andrew J. Syder, Jill Wykosky, Brian A. Perrino

**Affiliations:** 1 Department of Physiology and Cell Biology, University of Nevada, Reno School of Medicine, Reno, NV USA; 2 Bioinformatics, BGI Group, Shenzhen, Guangdon China; 3 Leonard Davis School of Gerontology, University of Southern California, Los Angeles, CA USA; 4 Gastroenterology Drug Discovery Unit, Takeda Pharmaceutical Company Limited, San Diego, CA USA

**Keywords:** Smooth muscle, Stomach, Mfge8, α8 integrin, Myosin light chain phosphatase, Actins

## Abstract

Coordinated gastric smooth muscle contraction is critical for proper digestion and is adversely affected by a number of gastric motility disorders. In this study we report that the secreted protein Mfge8 (milk fat globule-EGF factor 8) inhibits the contractile responses of human gastric antrum muscles to cholinergic stimuli by reducing the inhibitory phosphorylation of the MYPT1 (myosin phosphatase-targeting subunit (1) subunit of MLCP (myosin light chain phosphatase), resulting in reduced LC20 (smooth muscle myosin regulatory light chain (2) phosphorylation. Mfge8 reduced the agonist-induced increase in the F-actin/G-actin ratios of β-actin and γ-actin1. We show that endogenous Mfge8 is bound to its receptor, α8β1 integrin, in human gastric antrum muscles, suggesting that human gastric antrum muscle mechanical responses are regulated by Mfge8. The regulation of gastric antrum smooth muscles by Mfge8 and α8 integrin functions as a brake on gastric antrum mechanical activities. Further studies of the role of Mfge8 and α8 integrin in regulating gastric antrum function will likely reveal additional novel aspects of gastric smooth muscle motility mechanisms.

## Introduction

Digestion of ingested food by the stomach involves accommodation, chemical and mechanical disruption of solids into chyme, and controlled emptying into the duodenum. To carry out these functions, the stomach is comprised of functional anatomic regions with distinct motility patterns (Kong and Singh 2008; Janssen et al. 2011). The fundus relaxes to accommodate ingested food and then tonically contracts to move the contents into the distal stomach where the solids are reduced in size by peristaltic contractions. Gastric emptying is regulated by contractions of the antrum and the resistance provided by the pyloric canal. Healthy gastric function depends on properly coordinated motor activities of the proximal and distal stomach (Tack and Janssen 2010). Animal models have been studied for many years, but the regulatory mechanisms underlying the motor activities of the human stomach are not as well understood (Goyal et al. 2019; Tack et al. 2019).

Membrane depolarization of gastrointestinal (GI) smooth muscles triggers contraction by opening voltage‐dependent (L‐type) Ca^2+^ channels, non‐selective cation currents, and other mechanisms that contribute to the Ca^2+^ influx and the increase in [Ca^2+^]_i_ (Zhang et al. 2011; Sanders et al. 2012). The increase in [Ca^2+^]_i_ activates calmodulin‐dependent myosin light chain kinase (MLCK) to phosphorylate LC20 at S19 (pS19), stimulating myosin ATPase activity to generate cross‐bridge cycling and contraction (Somlyo and Somlyo 2003; He et al. 2008). Termination of the contractile signal decreases [Ca^2+^]_i_ by Ca^2+^ removal mechanisms, and inactivation of MLCK (Somlyo and Somlyo 1986; Somlyo and Himpens 1989). LC20 is then dephosphorylated by MLCP, leading to relaxation (Alessi et al. 1992; Paul et al. 2002). MLCP activity is inhibited by upstream kinase-dependent signaling pathways (Feng et al. 1999; Kitazawa et al. 2003; Ito et al. 2004). Phosphorylation of the protein kinase C- (PKC) potentiated phosphatase inhibitor protein-17 kDa (CPI-17) by PKC greatly increases its inhibition of MLCP (Eto et al. 1995; Hayashi et al. 2001). Phosphorylation of MYPT1 at T696 (human isoform numbering) inhibits MLCP activity (Matsumura and Hartshorne 2008; Grassie et al. 2011). Phosphorylation of MYPT1 T853 by Rho-associated coiled-coil protein kinase 2 (ROCK2) reduces the affinity of MLCP to myosin filaments in vitro (Velasco et al. 2002). However, ROCK2 phosphorylation of MYPT1 T853 does not appear to affect MLCP activity in vivo (He et al. 2013; Chen et al. 2015). In addition, expression of the MYPT1 T853A mutant does not affect agonist-induced LC20 phosphorylation and force development in bladder and ileum smooth muscles (Gao et al. 2013; Chen et al. 2015). Thus, although it is elevated by ROCK2 activation, MYPT1 T853 phosphorylation is not necessary for agonist-induced Ca^2+^ sensitization of smooth muscle (Gao et al. 2013; He et al. 2013; Chen et al. 2015). However, ROCK2 activity in smooth muscles is clearly required for Ca^2+^ sensitization and augmented contraction (Chen et al. 2015). Therefore, the level of MYPT1 T853 phosphorylation can be used as an indicator of myofilament Ca^2+^ sensitization in smooth muscles. Inhibiting MLCP while activating MLCK generates greater force by further increasing LC20 phosphorylation (Kitazawa et al. 1991; Mizuno et al. 2008). This phenomenon was termed “Ca^2+^ sensitization of the contractile apparatus,” to describe the increased Ca^2+^sensitivity of the contractile response (Somlyo and Somlyo 2003).

In addition to the actin filaments which interact with myosin thick filaments, smooth muscle cells contain a cortical actin cytoskeleton lying just under the plasma membrane which strengthens the membrane for the transmission of force to the extracellular matrix, and to enable the drastic morphological changes as smooth muscle cells shorten during contraction (Mehta and Gunst 1999; Gunst and Zhang 2008; Kim et al. 2008; Lehman and Morgan 2012). Smooth muscle cells can contract to 50% of their initial length, compared to only 20% for striated muscles (Widmaier et al. 2011). The cortical actin cytoskeleton must be flexible to allow these changes. It is now known that the dynamic reorganization of the cortical actin cytoskeleton also participates in force transduction, stiffness and adhesion, by increases in actin polymerization in response to contractile stimuli (Gunst and Zhang 2008; Kim et al. 2008; Lehman and Morgan 2012).

A novel mechanism regulating ROCK2-dependent myofilament Ca^2+^ sensitization in gastric smooth muscles has recently been described in murine gastric antrum muscles, involving the secreted protein Mfge8 (milk fat globule-EGF factor 8). (Khalifeh-Soltani et al. 2016). Mfge8 (or lactadherin) was initially identified as a principal component of the milk fat globule, a collection of membrane-encircled proteins and triglycerides that bud from the apical surface of mammary epithelia during lactation (Raymond et al. 2009). Mfge8 has since been shown to be ubiquitously expressed and to participate in a wide variety of cellular interactions, including phagocytosis of apoptotic lymphocytes and other apoptotic cells, sperm-egg adhesion, repair of intestinal mucosa, mammary gland branching morphogenesis, angiogenesis, attenuating inflammation, promoting wound healing, and enhancing tumorigenicity and cancer metastasis (Raymond et al. 2009; Li et al. 2013).

α8β1 integrins are RGD-binding integrins that were initially found to be critical for kidney morphogenesis where deletion of the α8 subunit leads to impaired recruitment of mesenchymal cells into epithelial structures (Müller et al. 1997; Humbert et al. 2014). It was recently shown that Mfge8 contains the RGD integrin binding sequence, and that Mfge8 is a ligand for α8β1 integrins (Khalifeh-Soltani et al. 2016). α8β1 integrins are also prominently expressed in smooth muscle and Mfge8 modulates smooth muscle contractile force (Kudo et al. 2013; Zargham et al. 2007; Zargham and Thilbault 2006; Schnapp et al. 1995). The binding of Mfge8 to α8β1 integrin heterodimers results in the inhibition of MYPT1 phosphorylation by ROCK2 and inhibition of antral contractility and gastric emptying (Khalifeh-Soltani et al. 2016). In contrast, in Mfge8^−/−^ mice, or α8 integrin^−/−^ mice, MYPT1 phosphorylation and antral contractility and gastric emptying are increased (Khalifeh-Soltani et al. 2016). These findings indicate that Mfge8 binding to α8β1 integrins acts as a “brake” on gastric muscle contractions and suggest that the endogenous level of gastric Mfge8 plays a role in regulating gastric motility. We have previously found that MYPT1 T853 is constitutively phosphorylated in human gastric smooth muscles, and is decreased by ROCK2 inhibition (Bhetwal et al. 2011, 2013a, b). However, whether Mfge8 regulates MYPT1 phosphorylation and the contractile responses of human gastric smooth muscles has not been reported. In this report, we show that, similar to mouse gastric antrum muscles, Mfge8 is present in human gastric antrum muscles and is constitutively bound to α8β1 integrin. We found that exogenously added Mfge8 inhibits the contractions evoked by electric field stimulation of cholinergic motor neurons, and the contractile responses to the cholinergic agonist carbachol (CCh). Exogenously added Mfge8 also reduced basal and CCh-evoked MYPT1 T696 and T853, and LC20 S19 phosphorylation levels, and inhibited the CCh-induced increase in cortical F-actin.

## Materials and methods

### Human stomach smooth muscles

The use of human resected stomach tissues was approved by the Human Subjects Research Committees at the Renown Regional Medical Center and the Biomedical Institutional Review Board at the University of Nevada, Reno, and was conducted in accordance with the Declaration of Helsinki (revised version, October 2008, Seoul, South Korea). All patients provided written informed consent. Resected stomach specimens were acquired immediately after surgery from patients undergoing sleeve gastrectomy. The resected stomach tissue was placed into ice-cold Krebs–Ringer buffer (KRB; composition (in mM): NaCl 118.5, KCl 4.5, MgCl_2_ 1.2, NaHCO_3_ 23.8, KH_2_PO4 1.2, dextrose 11.0, and CaCl_2_ 2.4; for transport to the laboratory. The gastric fundus region was identified by its bulbous appearance, and the gastric antrum region was identified by its narrow tapered shape. The resected stomach tissues were opened along the staples, laid out flat, and pinned to a Sylgard-lined dish containing oxygenated KRB. The mucosa and submucosa were removed by sharp dissection. Gastric antrum muscles were mapped and obtained from regions 13–16 (Rhee et al. 2011). Rectangular strips (∼4 mm × 10 mm × 2 mm) of full thickness muscle were used for the contractile studies and the protein phosphorylation studies. Larger strips snap-frozen in liquid N_2_ were used for differential centrifugation to obtain the ratios of filamentous (F)-actin to globular (G)-actin.

### Mechanical responses

Gastric antrum smooth muscle strips were attached to a Fort 10 isometric strain gauge (WPI, Sarasota, FL, USA), in parallel with the circular muscles, and pretreated with 2 µM neostigmine for 10 min at 37 °C in oxygenated KRB, and three 1 min washes with KRB, to remove any residual curariform neuromuscular paralytics (Li et al. 2018). Contractions were measured in static myobaths with oxygenated Krebs bubbled with 97% O_2–_3% CO_2_ at 37 °C, the pH of KRB was 7.3–7.4). Each strip was stretched to an initial resting force of ~ 0.8 g and then equilibrated for 45 min-60 min in 37 °C oxygenated KRB. To measure the contractile responses to KCl or CCh, the muscle strips were incubated with 0.3 µM tetrodotoxin to eliminate motor neuron activity. To measure contractile responses in response to electrical field stimulation, the muscle strips were incubated with LNNA and MRS2500 to eliminate nitrergic and purinergic motor neuron activity (Bhetwal et al. 2013b). Contractile activity was acquired and analyzed with AcqKnowledge 3.2.7 software (BIOPAC Systems, www.biopac.com).

### Automated capillary electrophoresis and immunodetection with Wes Simple Western

For automated capillary electrophoresis and Western blotting by Wes, the muscles were submerged into ice-cold acetone/10 µM dithiothreitol (DTT)/10% (w/v) trichloroacetic acid for 2 min, snap-frozen in liquid N_2_, and stored at − 80 °C for subsequent Wes analysis (Li et al. 2018; Xie et al. 2018). Muscles were washed in ice‐cold‐acetone–10 µM DTT for 1 min, 3 times, followed by a 1 min wash in ice‐cold lysis buffer (mM: 50 Tris–HCl pH 8.0, 60 β‐glycerophosphate, 100 NaF, 2 EGTA, 25 sodium pyrophosphate, 1 DTT, 0.5% NP‐40, 0.2% sodium dodecyl sulfate and protease inhibitors (Bhetwal et al. 2011). Tissues were homogenized in 0.5 ml lysis buffer in a Bullet Blender (0.01% anti‐foam C, one stainless steel bead per tube, speed 6, 5 min), then centrifuged at 16,000×*g*, for 10 min at 4 °C. Supernatants were stored at − 80 °C. Protein concentrations of the supernatants were determined by the Bradford assay using bovine γ‐globulin as the standard. Protein expression and phosphorylation levels were measured and analyzed according to the Wes User Guide using a Wes Simple Western instrument from ProteinSimple (www.proteinsimple.com). The protein samples were mixed with the fluorescent 5X master mix (ProteinSimple) and then heated at 95 °C for 5 min. Boiled samples, biotinylated protein ladder, blocking buffer, primary antibodies, ProteinSimple horseradish peroxidase‐conjugated anti‐rabbit or anti‐mouse secondary antibodies, luminol‐peroxide and wash buffer were loaded into the Wes plate (Wes 12–230 kDa Pre‐filled Plates with Split Buffer, ProteinSimple). The plates and capillary cartridges were loaded into the Wes instrument, and protein separation, antibody incubation and imaging were performed using default parameters. Compass software (ProteinSimple) was used to acquire the data, and to generate image reconstruction and chemiluminescence signal intensities. The protein and phosphorylation levels are expressed as the area of the peak chemiluminescence intensity. The following primary antibodies were used for Wes analysis: rabbit anti-γ-actin1, rabbit anti-enteric γ-actin2, GTX55849, www.genetex.com; mouse-anti β-actin, mouse anti-integrin-α8, MAB6194, www.rndsystems.com; rabbit anti-integrin-β1, sc-8978; rabbit anti‐LC20, sc‐15,370; www.scbt.com; rabbit anti-Mfge8, HPA002807, www.sigmaaldrich.com; rabbit anti‐MYPT1 (PPP1R12A), sc‐25,618; rabbit anti‐pT696‐MYPT1, sc‐17,556‐R; rabbit anti‐pT853‐MYPT1, sc‐17,432‐R; rabbit anti-pS19-LC20, PA5-17,726, www.thermofisher.com.

### Immunofluorescence and in situ proximity ligation assay (PLA)

For both immunofluorescence and isPLA the gastric antrum smooth muscle strips were fixed with 4% paraformaldeyde in PBS, and then cryo-protected with PBS/30% sucrose at 4 °C, embedded in OCT, and frozen at − 80 °C (Xie and Perrino 2019). The blocks were cut using a microtome into 10 µm sections and placed onto Vectabond (SP-1800) coated glass slides (Fisherbrand Superfrost Plus Microscope Slides, 12–550-15). After 20 min microwave heat-induced antigen retrieval in Tris–EDTA buffer (10 mM Tris base, 1 mM EDTA solution, 0.05% Tween 20, pH 9.0), the slides were permeabilized and blocked with PBS containing 0.2% Tween-20 and 1% BSA for 10 min at room temperature. The slides were then incubated overnight at 4 °C with the appropriate primary antibody as indicated below. Immunofluorescent labeling was performed with the appropriate Alexa-488 or Alexa-594 conjugated secondary antibody (Cell Signaling Technology, www.cellsignal.com) against the primary antibody (1:500 for 30 min at room temperature in PBS). isPLA was performed according to the manufacturer's instructions using the Duolink In Situ Detection Reagents Red DUO92008 (Sigma-Aldrich, Olink Bioscience, Sweden, www.sigmaaldrich.com) (Xie and Perrino 2019). The muscle sections were incubated with each primary antibody (1:400 dilution) sequentially for 1 h at room temperature. The slides were then incubated with the appropriate PLA probes (diluted 1:5 in PBS containing 0.05% Tween-20 and 3% bovine serum albumin) in a pre-heated humidified chamber at 37 °C for 1 h, followed by the ligation (30 min, 37 °C) and amplification (100 min, 37 °C) reactions. Mounting medium with DAPI was used to label nuclei blue. It has been reported that the number of PLA signals can decrease as kits get older (Ulke-Lemée et al. 2015). We did not experience any differences in the PLA results as the kits aged. However, control and treated muscle sections were compared using Duolink Detection kits from the same lot number prior to the lot expiration date. The following antibodies were used for isPLA: mouse anti-integrin-α8, MAB6194, www.rndsystems.com; rabbit anti-integrin-β1, sc-8978, www.scbt.com; rabbit anti-Mfge8, HPA002807, www.sigmaaldrich.com; rabbit anti-enteric γ-actin, GTX55849, www.genetex.com.

### Confocal microscopy and image acquisition

The slides were examined using an LSM510 Meta (Zeiss, www.zeiss.com) or Fluoview FV1000 confocal microscope (Olympus,www.olympus-lifescience.com) (Xie and Perrino 2019). Confocal micrographs are digital composites of the Z-series of scans (1 µm optical sections of 10 µm thick sections). Settings were fixed at the beginning of both acquisition and analysis steps and were unchanged. Brightness and contrast were slightly adjusted after merging. Final images were constructed using FV10-ASW 2.1 software (Olympus). Each image is representative of labeling experiments from 3 sections from 3 gastric antrum muscles. Scale bars, 10 µm.

### Differential ultracentrifugation of homogenates for filamentous (F)- and globular (G)-actin ratios

The protocol was as described (Kim et al. 2008; Bhetwal et al. 2013a), with some modifications. Muscle strips were homogenized in 1 ml 37 °C fractionation buffer (50 mM PIPES (pH 6.9), 50 mM NaCl, 5 mM MgCl2, 5 mM EGTA, 5% (vol/vol) glycerol, 0.1% Nonidet P-40 (NP-40), 0.1% Triton X-100, 0.1% Tween 20, 100 mM ATP, 1 mM dithiothreitol, 0.001% antifoam C, and a protease inhibitor tablet) in a Bullet Blender (two stainless steel bead per tube, speed 6, until a uniform homogenate was obtained), then centrifuged at 16,000×*g*, for 10 min at 32 °C. The supernatants were transferred to a prewarmed (37 °C) ultracentrifuge rotor and spun at 100, 000 g for 1 h at 37 °C to separate the globular G-actin (supernatant) and filamentous F-actin (pellet) fractions. The pellets were resuspended in 200 µl of ice-cold lysis buffer. Both fractions were stored at − 80 °C. Protein concentrations were determined by the Bradford assay using bovine γ‐globulin as the standard.

### Data and statistical analysis

Contractile responses were compared by measuring the area under the curve (AUC) of each peak including the contribution of basal tone (integral, grams × seconds) divided by time (seconds), per cross‐sectional area (cm^2^) of the smooth muscles, using Acknowledge. The average peak responses (mean (SD)) were calculated using Prism, and significance was determined by *t* test using Prism with *P* < 0.05 considered as significant. Graphs were generated using Prism. The area of the peak chemiluminescence intensity values of the protein bands were calculated by Compass software. The chemiluminescence intensity values of pT696, pT853, and pS19 were divided by the total MYPT1, and LC20 chemiluminescence intensity values from the same sample, respectively, to obtain the ratio of phosphorylated protein to total protein. The ratios were normalized to 1 for unstimulated muscles and all ratios were subsequently analyzed by non‐parametric repeated tests of ANOVA using Prism 7.01 software (GraphPad Software, www.graphpad.com), and are expressed as the means ± SD. Student's t test was used to measure significance and P < 0.05 is considered significant. The digital lane views (bitmaps) of the immunodetected protein bands were generated by Compass software, with each lane corresponding to an individual capillary tube. The PLA figures were created from the digitized data using Adobe Photoshop Version 12.0.3. Fiji software was used to count PLA spots (Xie and Perrino 2019). Graphs were generated using GraphPad/Prism.

### Drugs and reagents

Recombinant human Mfge8 and recombinant human laminin subunit alpha-1 were purchased from R&D Systems, www.rndsystems.com; atropine and tetrodotoxin were obtained from EMD Millipore, www.emdmillipore.com; and MRS2500 was purchased from Tocris Bioscience, www.tocris.com. All other reagents and chemicals purchased were of analytical grade or better.

## Results

### Human gastric antrum muscles express Mfge8, α8 integrin, and β1 integrin

**Fig. 1:**
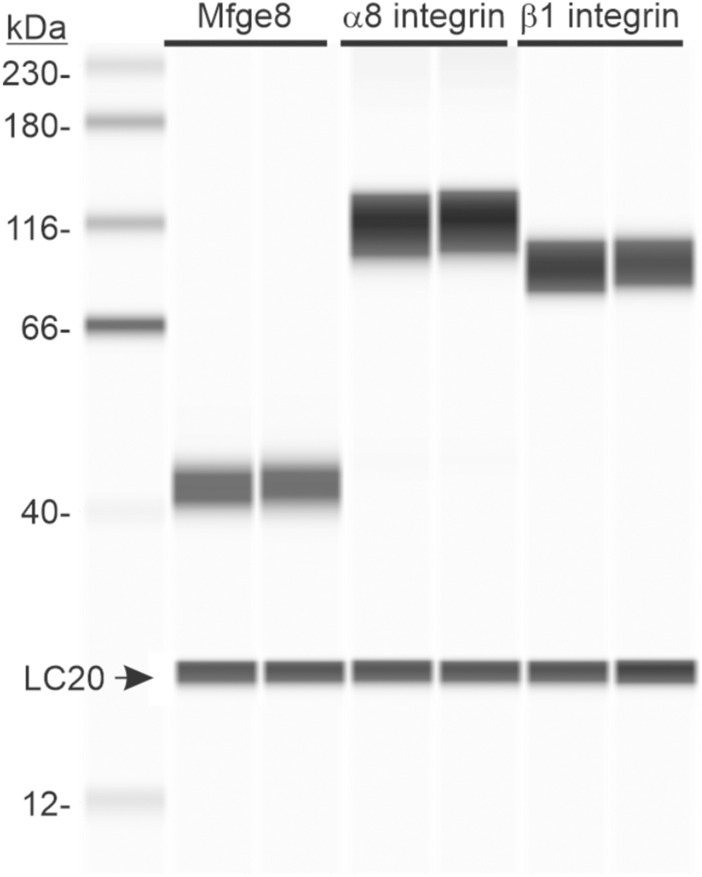
Mfge8, α8 integrin, and β1 integrin are expressed in human gastric antrum smooth muscles. Representative Wes image of Mfge8, α8 integrin, and β1 integrin proteins in gastric antrum smooth muscle by chemiluminescence immunodetection using anti- Mfge8 (100X dilution), α8 integrin (100X dilution), and β1 integrin (100X dilution) antibodies in duplicate as described in the Methods. 5.0 µg lysate protein per lane. Anti-LC20 (1:500 dilution) immunodetection was used as the loading control. (n = 5.)

Since Mfge8 and α8 integrin expression in human gastric antrum muscles has not been reported, we examined homogenates of human gastric antrum muscles for Mfge8 and α8 integrin protein expression, along with β1 integrin protein expression. Similar to murine gastric antrum muscles, human gastric antrum muscles express Mfge8 (43 kDa), α8 integrin (118 kDa), and β1 integrin (89 kDa), as shown by the Wes analysis of human gastric antrum muscle lysates in Fig. 1.

### Human gastric antrum muscles contain α8β1 integrin heterodimers and Mfge8

**Fig. 2:**
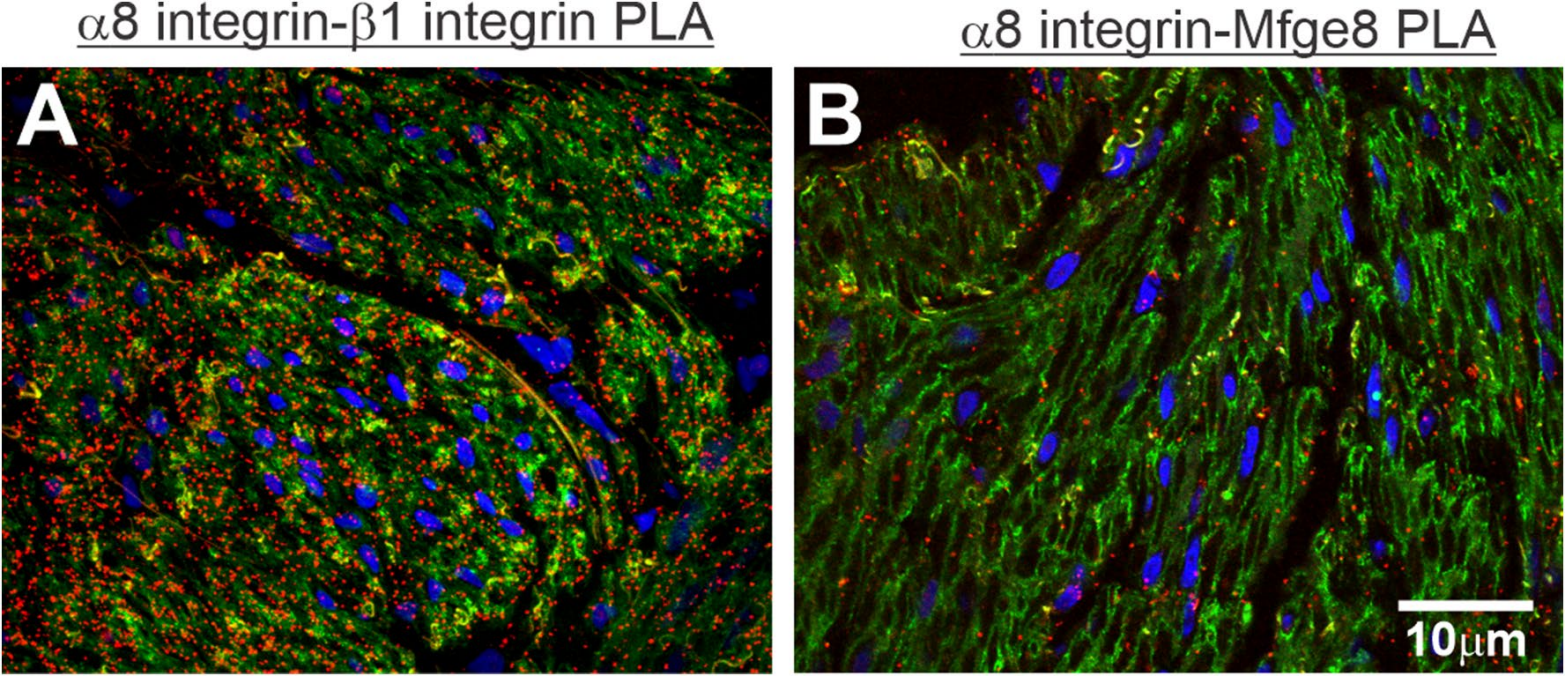
α8β1 integrin heterodimers and Mfge8 interactions with α8 integrin in human gastric antrum smooth muscle shown by in situ PLA. Representative confocal microscopy images from gastric antrum smooth muscle sections. **A** Section immunostained with enteric γ-actin (green), and then probed with anti- α8 integrin and β1 integrin antibodies for PLA immunostaining (red spots). **B** Section immunostained with β1 integrin (green), and then probed with anti- Mfge8 and α8 integrin antibodies for PLA immunostaining (red spots). Cell nuclei were stained with DAPI (blue). (n = 5.). (Color figure online)

Because it was reported by Khalifeh-Soltani et al. (2016) that Mfge8 binds to α8 integrin in α8β1 integrin heterodimers in murine gastric antrum muscles, we used in situ PLA to determine whether Mfge8 binds to α8 integrin in α8β1 integrin heterodimers in human gastric antrum muscles. We also immunostained enteric γ-actin2 to localize smooth muscles cells in the antrum smooth muscle sections. The PLA results and enteric γ-actin immunostaining in Fig. 2A show that a8β1 integrin heterodimers are present in human gastric antrum smooth muscles. We then carried out in situ PLA using anti α8 integrin and anti Mfge8 antibodies to determine whether human gastric antrum smooth muscles contain Mfge8 bound to α8 integrin. We also immunostained β1 integrin to localize smooth muscle cell plasma membranes in the antrum smooth muscle sections. The PLA results and β1 integrin immunostaining in Fig. 2B show that Mfge8 is likely bound to α8 integrin in human gastric antrum smooth muscles. Particle analysis of the PLA spots using FIJI suggests that the number of a8b1 heterodimers (954 ± 78 spots per µm^2^) is significantly greater than the number of a8 integrins bound to Mfge8 (318 ± 47 spots per µm^2^) (P < 0.01, n = 5).

### Exogenously added Mfge8 inhibits CCh-evoked contractions of human gastric antrum muscles

**Fig. 3:**
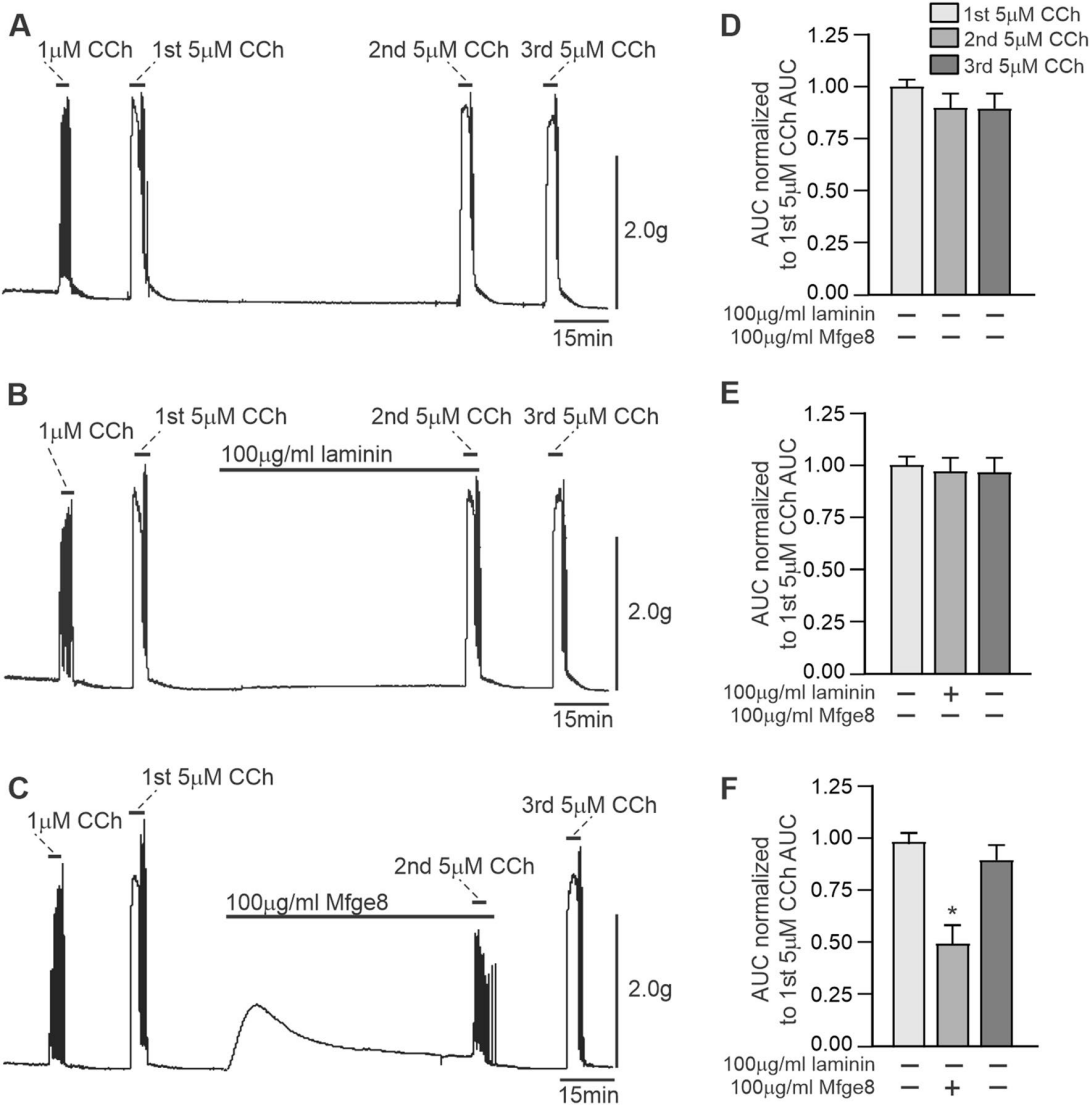
Exogenously added Mfge8 inhibits CCh-evoked contractions of human gastric antrum smooth muscle. Representative tension recordings of the contractile responses to 5 µM CCh alone (**A**), or in the presence of 100 µg/ml laminin (**B**), or 100 µg/ml Mfge8 (**C**). Summarized data of the areas under the curve of each contractile response (**D**–**F**). (n = 6; 2 muscle strips from 3 gastric antrums; Averages are ± SD, *P < 0.05)

We next determined if Mfge8 can regulate human gastric antrum muscle contractile responses. Figure 3 shows the isometric contractile responses of human gastric antrum muscle strips to the cholinergic agonist CCh. CCh at concentrations of 1 µM and 5 µM dose-dependently increased the force of contractions, as shown in the contractile recordings and the summarized data. After washout of CCh, Mfge8 was added to the myobaths at a concentration of 100 µg/ml, and incubated with the muscle strips for 90 min. Laminin was added to separate myobaths at a concentration of 100 µg/ml, as a negative control integrin RGD-binding protein (Zheng and Leftheris 2020). As shown in Fig. 3B and C, the addition of Mfge8 cause a transient contraction of the muscle strips, while laminin had no effect upon addition to the myobath. As shown in Fig. 3A and D, the contractile responses to 5 µM CCh 90 min after the first 5 µM CCh-evoked contraction were unchanged. Similarly, after incubation with laminin for 90 min, Fig. 3B and E show that the contractile responses of human gastric antrum muscle strips to 5 µM CCh were similar to the first 5 µM CCh-evoked contraction. In contrast, Fig. 3C and F show that compared to the first 5 µM CCh-evoked contraction, the contractile response of human gastric antrum muscle strips to 5 µM CCh was significantly decreased (~ 50%) by incubation with Mfge8 for 90 min. In addition, Fig. 3C and F show that the contractile responses of the muscle strips to 5 µM CCh recovered following washout of Mfge8, as indicated by the increase in the AUC.

### Exogenously added Mfge8 inhibits MYPT1 and LC20 phosphorylation in human gastric antrum muscles

**Fig. 4:**
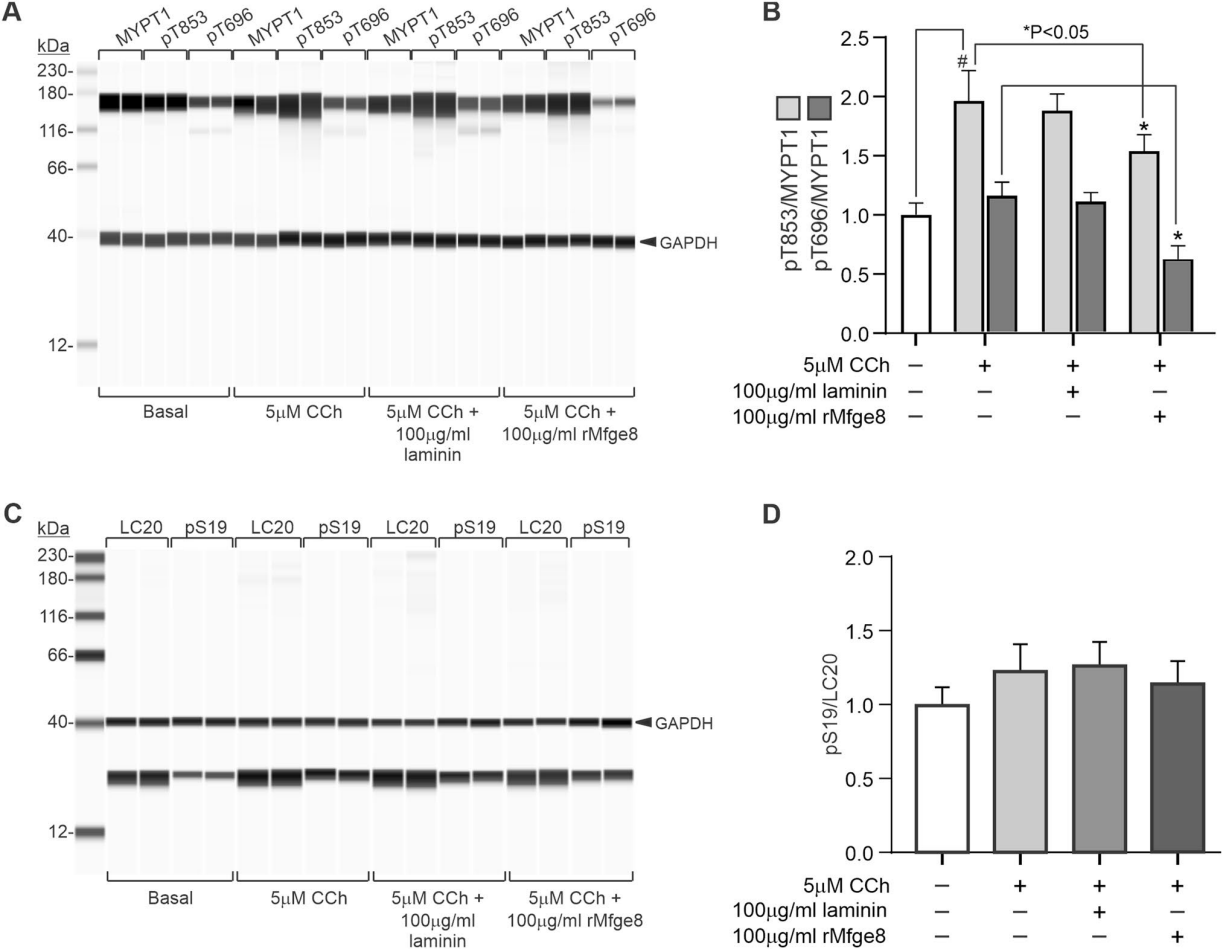
Exogenously added Mfge8 inhibits CCh-evoked phosphorylation of MYPT1 in human gastric antrum smooth muscles. **A** Representative Wes analysis of MYPT1 T853 and T696 phosphorylation by 5 µM CCh alone, or in the presence of 100 µg/ml laminin, or 100 µg/ml Mfge8. **B** Summary of the effects of 5 µM CCh alone, or in the presence of 100 µg/ml laminin, or 100 µg/ml Mfge8 on MYPT1 T853 and T696 phosphorylation. **C** Representative Wes analysis of LC20 S19 phosphorylation by 5 µM CCh alone, or in the presence of 100 µg/ml laminin, or 100 µg/ml Mfge8. **D** Summary of the effects of 5 µM CCh alone, or in the presence of 100 µg/ml laminin, or 100 µg/ml Mfge8 on LC20 S19 phosphorylation. GAPDH immunodetection was used as the loading control. (n = 6; 2 muscle strips from 3 gastric antrums, Averages are ± SD, ^#^Significantly different from Control; *Significantly different from CCh, ^#*^P < 0.01)

It was previously determined that Mfge8 inhibits murine gastric antrum muscle contractions by inhibiting MYPT1 pT696 phosphorylation, resulting in decreased LC20 phosphorylation (Khalifeh-Soltani et al. 2016). Since we found that Mfge8 inhibits human gastric antrum muscle contractions, we examined whether CCh-evoked MYPT1 and LC20 phosphorylation are inhibited by Mfge8. As shown in Fig. 4A and B, 5 min treatment with 5 µM CCh significantly increased MYPT1 T853 phosphorylation by approximately twofold. MYPT1 T696 phosphorylation was increased, but this increase was not significant. Incubation with laminin for 90 min had no effect on the CCh-evoked increase in MYPT1 T853 phosphorylation and did not affect T696 phosphorylation. However, Fig. 4A and B show that the CCh-evoked increase in MYPT1 T853 phosphorylation was significantly inhibited (~ 30% reduction) by incubation with Mfge8 for 90 min, and MYPT1 pT696 phosphorylation was significantly reduced (~ 30% reduction). Figure 4C and D show that LC20 S19 phosphorylation was consistently increased by CCh treatment, but this increase was not statistically significant. Laminin had no effect on the increase in LC20 S19 phosphorylation (Fig. 4C, D). In contrast, the CCh-evoked increase in LC20 S19 phosphorylation was inhibited by incubation with Mfge8 for 90 min, but this decrease was not statistically significant.

**Fig. 5:**
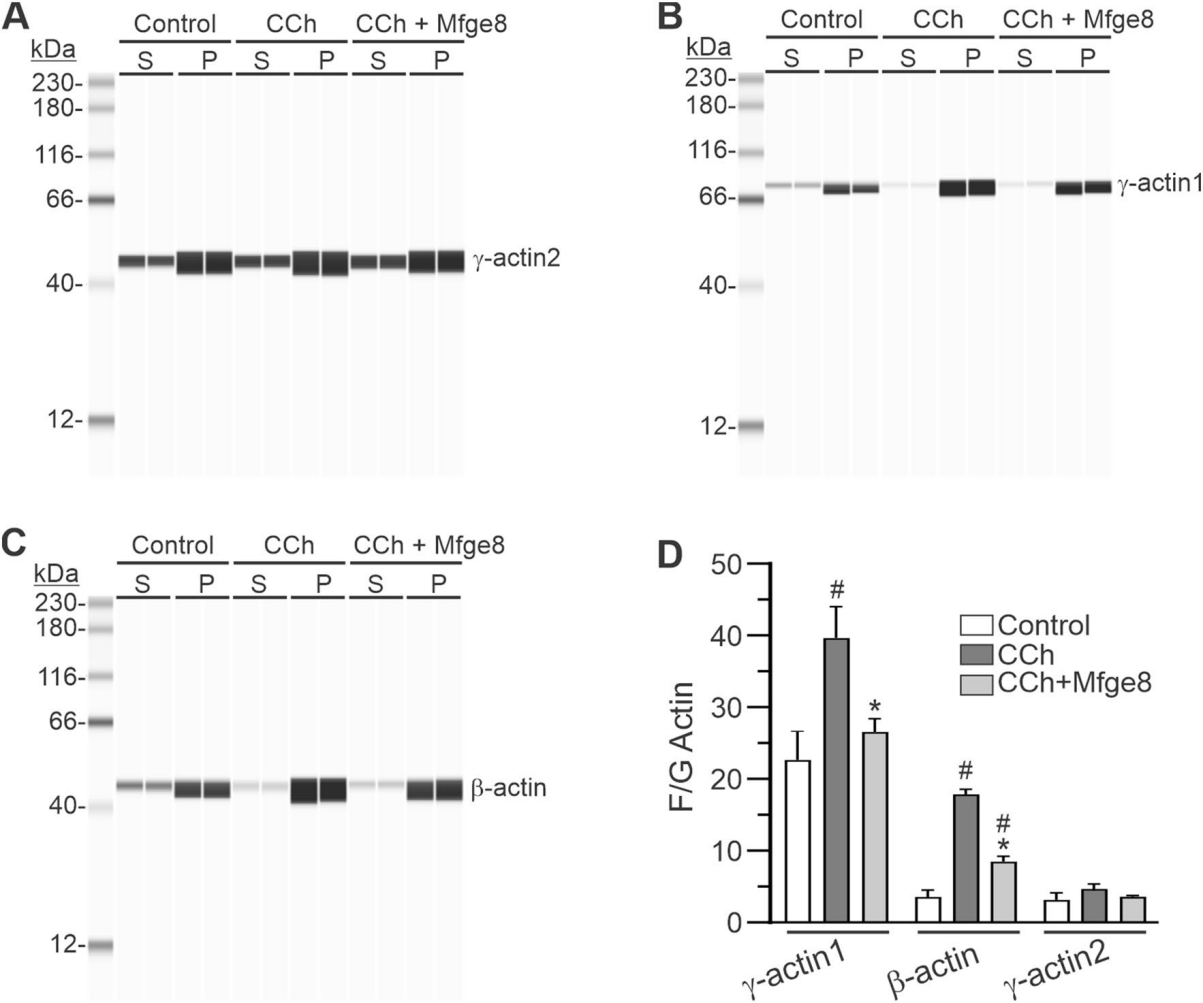
Exogenously added Mfge8 inhibits the CCh-evoked increase in actin F/G ratios in human gastric antrum smooth muscles. Differential ultracentrifugation of muscle homogenates indicates Mfge8 inhibits the CCh-induced increase in filamentous (F-actin) to globular (G-actin) ratios (F/G ratio). Representative Wes analyses of γ-actin2 (**A**), γ-actin1 (**B**), and β-actin (**C**) levels in the 100,000×*g* supernatant (S, G-actin) and pellet (P, F-actin). **D** F/G Actin ratios from the chemiluminescence intensity values of the actin bands. (n = 4 gastric antrums, Averages are ± SD, ^#^Significantly different from Control; *Significantly different from CCh, ^#*^P < 0.01)

### Exogenously added Mfge8 inhibits the increase in F-actin evoked by CCh stimulation of human gastric antrum muscles

Agonist-induced actin polymerization associated with the cortical actin cytoskeleton in smooth muscle cells occurs during a contractile stimulus (Mehta and Gunst 1999; Gunst and Zhang 2008; Kim et al. 2008; Lehman and Morgan 2012). Therefore, we investigated the effects of exogenously added Mfge8 on the actin F/G ratios in CCh-stimulated muscles. In human gastric antrum muscles, the contractile enteric γ-actin2 isoform (ACTG2) is the most highly expressed actin isoform (Table 1), comprising approximately 80% of the total actin. The cytoskeletal β-actin (ACTB) and γ-actin1 (ACTG1) isoforms are present at much lower amounts, with β-actin isoform expression (~ 17% of total) almost tenfold higher than γ-actin1 isoform expression (~ 0.2% of total) (Table 1). As shown in Fig. 5D, the ratio of F/G actin for γ-actin1 is a little over 20:1, approximately fourfold higher than the F/G actin ratios of β-actin and γ-actin2. CCh stimulation increased the β-actin F/G ratio by almost fourfold, and increased the γ-actin1 F/G ratio by almost twofold, but only slightly, and not significantly, increased the F/G ratio of enteric γ-actin2. Exogenously added Mfge8 significantly inhibited the CCh-evoked increase in the F/G ratios of β-actin (~ 50%) and γ-actin1 (~ 30%), and had a slight effect on the CCh-evoked increase in the F/G ratio of γ-actin2.

**Table 1:** Protein expression levels of actin isoforms in human gastric antrum muscles

γ-actin2	β-actin	γ-actin1
1028 ± 153	205 ± 79	2.6 ± 0.7

Data are mean ± SD and expressed as chemiluminescence intensity per ng protein (n = 3 gastric antrum muscles). One way ANOVA indicates significant differences between the means (P < 0.05)

## Discussion

It was previously reported that Mfge8 inhibits antral muscle contractions and slows gastrointestinal motility in mice by specifically binding to α8 integrin in α8β1 integrin heterodimers, resulting in reduced phosphorylation of the inhibitory MYPT1 subunit of MLCP, and consequentially reduced LC20 phosphorylation (Khalifeh-Soltani et al. 2016). In addition, either smooth muscle-specific deletion of Mfge8 or α8 integrin resulted in an increase in gastric antral contractile force, more rapid gastric emptying, and faster small intestinal transit times (Khalifeh-Soltani et al. 2016). These findings revealed a novel inhibitory mechanism regulating gastric antrum function, raising the question as to whether a similar mechanism is involved in regulating human gastric antrum smooth muscle contractile responses. The expression of Mfge8 or α8 integrin in human gastric antrum muscles has not been described previously, thus in this study we determined that both Mfge8 and α8β1 integrin heterodimers are present in human gastric antrum muscles, and that Mfge8 is bound to α8β1 integrin heterodimers. We also show that exogenously added Mfgfe8 inhibits the contractile responses of human gastric antrum muscles to exogenous and endogenous cholinergic stimuli. This inhibition of contraction was accompanied by inhibition of MYPT1 and LC20 phosphorylation, supporting a novel role for α8β1 integrins and Mfge8 in regulating human gastric motility by attenuating MYPT1 phosphorylation. We used in situ PLA to demonstrate the interaction between Mfge8 and α8 integrin. We were not able to examine the effects of abrogating the binding of Mfge8 to α8β1 integrins because there is no inhibitor of Mfge8 binding to α8β1 integrins available. However, adding Mfge8 protein to muscle strips in the myobaths significantly inhibited the contractile responses to the cholinergic agonist CCh or to EFS-evoked cholinergic neurotransmission. These findings suggest that there are α8β1 integrins not occupied by Mfge8, and that increases in Mfge8 could further inhibit gastric antrum muscle contraction. The results in Fig. 2 showing that there are significantly greater α8β1 integrin heterodimer PLA signals than the Mfge8-α8 integrin PLA signals support this conclusion. These findings strongly suggest that Mfge8 is involved in the regulation of human gastric antrum muscle mechanical responses.

Agonist-stimulated actin polymerization, as shown by an increase in the F/G actin ratio, has been shown to correlate with an increase in the contractile force generated in vascular and airway smooth muscle (Mehta and Gunst 1999; Gunst and Zhang 2008; Kim et al. 2008; Lehman and Morgan 2012). In vascular smooth muscle, the cytoskeletal actin isoform γ-actin1, which is primarily localized to the sub-membranous actin cortex, is most sensitive to G-actin to F-actin conversion in response to vasoconstrictors, reflecting changes in polymerization/ depolymerization (Kim et al. 2008). Similarly, we found that CCh treatment increased the F/G ratios of the two cytoskeletal actin isoforms, γ-actin1 and β-actin, in human gastric antrum muscles. However, we found that CCh-stimulation induced a larger increase in the β-actin F/G ratio (~ fourfold) than the γ-actin1 ratio (~ twofold). This may be due to the finding that β-actin is much more highly expressed than γ-actin1 in human gastric antrum muscles, and thus would comprise the bulk of the actin cortical cytoskeleton. The F/G ratio of enteric γ-actin2 did not significantly change during CCh stimulation, likely due to its primary localization within the contractile actin filaments (McHugh and Lessard 1988). As expected, enteric γ-actin2 comprises the bulk of total actin expressed in gastric antrum muscles, followed by β-actin and γ-actin1 at much lower amounts.

Mfge8 (originally named lactadherin) was first identified in breast milk, having antimicrobial and antiviral effects, and playing an important role in immune defense as a secreted immune system molecule (Stubbs et al. 1990; Atabai et al. 2005). Mfge8 is now known to be a ubiquitously expressed multifunctional protein belonging to the family of secreted integrin-binding glycoproteins containing the RGD integrin-binding motif (Raymond et al. 2009). The most well known role for α8β1 integrin is in kidney morphogenesis where deletion of α8 integrin leads to impaired recruitment of mesenchymal cells into epithelial structures (Müller et al. 1997; Humbert et al. 2014). α8 integrin is a member of the RGD-binding integrin family that is prominently expressed in smooth muscle coupled to β1 integrin (Schnapp et al. 1995; Zargham and Thibault 2006; Zargham et al. 2007). Previous work has shown the expression of α8 integrin in both vascular and visceral smooth muscle, as well as the muscularis mucosa of the GI tract (Schnapp et al. 1995). In vitro studies suggest that α8 integrin promotes smooth muscle differentiation, and maintains vascular smooth muscle in a differentiated, contractile, non-migratory phenotype (Zargham and Thibault 2006; Zhang et al. 2016). Mfge8 and α8 integrin also modulate smooth muscle contractile force. In Mfge8^−/−^ mice, or α8 integrin^−/−^ mice, airway and jejunal smooth muscle contraction are enhanced in response to contractile agonists after these muscle beds have been exposed to inflammatory cytokines but not under basal conditions (Kudo et al. 2013; Khalifeh-Soltani et al. 2016, 2018). Whether the origin of Mfge8 in gastric muscles is from circulating Mfge8 or is locally secreted is unclear. Mfge8 can reach the gastric antrum smooth muscle layer by oral gavage, but it is not clear how Mfge8 reaches the gastric antrum smooth muscle layer, or how widespread the distribution of Mfge8 is after oral administration (Khalifeh-Soltani et al. 2016). Determining the source of Mfge8 present in gastric muscle tissues is an important issue to address in future studies of gastric motility regulatory mechanisms.

Elevations in cytosolic Ca^2+^ directly promote smooth muscle contraction by Ca^2+^/calmodulin activation of MLCK and phosphorylation of LC20 (Somlyo and Somlyo 2003). Rho kinase and PKC activities contribute to MLCK activity by phosphorylating the regulatory subunits of MLCP to promote LC20 phosphorylation and increase the myofilament sensitivity to Ca^2+^ (Perrino 2016). In addition, a number of studies have provided evidence that dynamic changes to the actin cytoskeleton play an important role in smooth muscle contraction (Mehta and Gunst 1999; Zhang et al. 2018). This remodeling process appears to facilitate the polymerization of cortical cytoskeletal actin filaments and increase the stability of focal adhesions in the membrane, allowing for the force generated by myofilament activation to be transmitted to the connective tissue of the extracellular matrix (Zheng et al. 1998; Mills et al. 2015). Tyrosine phosphorylation of protein tyrosine kinase 2 β (Pyk2) and focal adhesion kinase (FAK), along with the recruitment of other integrin‐associated proteins to focal adhesions, occurs during contraction and force development (Gerthoffer and Gunst 2001). In addition, we found that FAK also promotes gastric smooth muscle contraction by activation of the PKC-CPI-17 Ca^2+^ sensitization pathway (Xie et al. 2018).

In summary, in this study we report that the secreted protein Mfge8 inhibits the contractile responses of human gastric antrum muscles to cholinergic stimuli by reducing the inhibitory phosphorylation of the MYPT1 subunit of MLCP, resulting in reduced LC20 phosphorylation. We found that endogenous Mfge8 is bound to its receptor, α8β1 integrin, in human gastric antrum muscles, and that exogenously added Mfge8 inhibits CCh-evoked contraction, suggesting that human gastric antrum muscle mechanical responses are regulated by Mfge8. These findings, and the findings of Khalifeh-Soltani et al. 2016, reveal an additional pathway regulating the contractile responses of smooth muscles. The regulation of gastric antrum smooth muscles by Mfge8 and α8 integrin opposes the prokinetic actions of MLCK activation, MLCP inhibition, and actin cytoskeleton remodeling. In this regard, Mfge8 α8 integrin signaling seems to function as a brake on gastric antrum mechanical activities, and suggest that disrupting Mfge8 binding to α8β1 integrins in gastric smooth muscles may improve or reverse abnormal gastric antrum muscle mechanical responses associated with gastric motility disorders. Further studies of the role of Mfge8 and α8 integrin in regulating gastric antrum function will likely reveal additional novel aspects of gastric smooth muscle motility mechanisms.

## Data Availability

The datasets generated during and/or analyzed during the current study are available from the corresponding author on reasonable request.
